# Critical Electrospinning Parameters for Synthesis Control of Stabilized Polyacrylonitrile Nanofibers

**DOI:** 10.3390/nano13192648

**Published:** 2023-09-26

**Authors:** Juan Emmanuel Ruiz Rocha, Karla Rebeca Moreno Tovar, Ricardo Navarro Mendoza, Silvia Gutiérrez Granados, Sara Cavaliere, Domitille Giaume, Philippe Barboux, Jesús Salvador Jaime Ferrer

**Affiliations:** 1 Departamento de Química, División de Ciencias Naturales y Exactas, Universidad de Guanajuato, Pueblito de Rocha s/n, Guanajuato 36040, Mexico; je.ruiz-rocha@chimieparistech.psl.eu (J.E.R.R.); navarrm@ugto.mx (R.N.M.);; 2 Chimie ParisTech, PSL University, Institut de Recherche de Chimie Paris, Centre National de la Recherche Scientifique, 75005 Paris, France; domitille.giaume@chimieparistech.psl.eu (D.G.); philippe.barboux@chimieparistech.psl.eu (P.B.); 3 ICGM, University Montpellier, CNRS, ENSCM, CEDEX 5, 34095 Montpellier, France; 4 CIATEC A.C., Centro de Innovación Aplicada en Tecnología Competitiva, Omega 201, Industrial Delta, GTO, León 37545, Mexico

**Keywords:** electrospinning, polyacrylonitrile, stabilization, morphology

## Abstract

Polyacrylonitrile (PAN) fibers are widely used as precursors in the manufacture of high-conducting and mechanically resistant carbon fibers. The modulation of such fibers is carried out through electrospinning. In this work, we show the production and control of the morphology of nanometric-range PAN fibers for their potential use as precursors for high-electrical-conductivity carbon fibers. PAN samples dissolved in dimethylformamide (DMF) were prepared at 6, 10, and 12% *w*/*w*, at 15 and 25 kV. The impact of the rotation of the collector drum at 100, 300, and 500 RPM was also studied. It was found that the percentage of PAN in the solution proportionally affects the diameter of the fibers and that the preparation potential affects the morphology. The rotation speed, when increased, decreases the diameter, and it has a negative impact on the morphology. Fibers prepared with 6% *w*/*w* at 15 kV and 500 RPM show 90 nm diameters, the smallest diameter of all the samples.

## 1. Introduction

Carbon fiber formation was first observed in the 1950s during the pyrolysis of hydrocarbons [1]. However, it was not until the 1990s that Iijima et al. [2] discovered cylindrical carbon structures with diameters in the range of 10–500 nanometers, hence the name “carbon nanofibers” (CNFs) [3]. These structures were distinct from other flat or spherical carbon-based materials [4]. Research on CNFs has rapidly evolved, focusing on understanding their properties and developing methods to synthetize them.

Such key properties include high strength and stiffness, high surface area, high electrical conductivity, chemical and thermal stability, low density, and biocompatibility [5]. Due to these properties, CNFs have potential applications in energy storage, catalysis, and sensors, and particularly in supercapacitors, lithium-ion batteries, and fuel cells [6,7,8]. Some examples reported by Poudel et al. include a study about assembling zinc–cobalt-hydroxide/ternary sulfide heterostructure on three-dimensional hollow porous CNFs [9]. Another study concerns confined zinc–magnesium–aluminum layered double hydroxide nanosheets on hollow porous CNFs with a capacitance of 3437 F cm^−2^ [10]. For environmental purposes, these were applied for the adsorption of the heavy metal ions Cr (VI) and Pb (II) using Co-Al layered double hydroxide loaded with hematite (α-Fe_2_O_3_) @ 3D porous CNF, reporting adsorptions of 400.40 mg g^−1^ and 426.76 mg g^−1^, respectively [11]. Carbon nanofibers issued by electrospinning can also be used as self-supported electrodes to be used directly in fuel cell cathodes [12].

The structure of CNFs, including their diameter, length, and degree of crystallinity, affects their properties [13]. CNFs with a smaller diameter have higher surface area-to-volume ratios and greater reactivity [14]. Longer CNFs have higher mechanical strength. Highly crystalline CNFs display better thermal and electrical conductivity [15,16].The structure of CNFs is determined by the synthesis method and the polymer used as a carbon source [17]. Common synthesis methods include chemical vapor deposition, electrospinning, arc discharge, laser ablation, and template-assisted synthesis [18].

The first report on the use of electrospinning to produce PAN nanofibers was published by Reneker and Chun in 1996 [19]; subsequently, this technique was widely used, especially with PAN as a precursor [20,21,22,23]. The polymer molecular weight, solution concentration, voltage, flow rate, spinneret-collector distance, and environmental factors influence the electrospinning process and the resulting fiber properties [24,25,26]. Other electrospun nanofiber applications are in environmental toxicant sensors or personal healthcare devices through conjugated copolymers (CCP) that can be used for sensing various environments, such as metal ions, pH, temperature, humidity, etc. [27]. Another example is an antiviral agent for personal protective equipment such as facemasks and surgical gowns composed by nanocomposites such as PAN polymer, ZnO nanoparticles, and Viroblock [28].

DMF is the most commonly used solvent to dissolve and electrospin PAN due to its good solubility, low viscosity, good conductivity, high boiling point, and availability [29]. The molecular weight of PAN can affect the fiber properties. Low-molecular-weight PAN may produce nanofibers with lower mechanical strength and thermal stability [30]. On the other hand, higher-molecular-weight PAN can produce nanofibers with better mechanical properties and improved thermal stability [31]. Polymeric solution concentration influences fiber formation; low concentrations result in discontinuous fibers and high concentrations in thick fibers [32]. Voltage to electrospin PAN is typically between 10 and 30 kV [33].

An optimal flow rate to electrospin PAN nanofibers lies in the range of 0.1–2 mL h^−1^ and varies depending on the specific processing parameters. At lower flow rates, the electrostatic force generated may not be sufficient to pull the polymer solution from the tip of the spinneret, resulting in a low production rate [34]. Spinneret-collector distance influences fiber morphology and alignment, typically in the range of 10–30 cm [35,36]. The rotation speed of the collector drum affects nanofiber properties. High speeds favor thin and aligned fibers. Temperature and humidity conditions during electrospinning can also impact the process and fiber properties [37], affecting solution viscosity and flow rate, and solvent evaporation and fiber distribution [32], respectively.

To enhance thermal stability and mechanical strength to allow further processing into CNF, PAN nanofibers must undergo a thermal treatment at temperatures between 250 and 270 °C, causing oxidation and cyclization [36,38]. The main reaction is an oxidation of the nitrile groups. The formation of polar functional groups in the fibers and of covalent bonds between adjacent nitrile groups in the polymer chains derive into the formation of ladder-like structures [39,40]. Carbonization in an inert gas atmosphere results in carbon nanofibers with tailored properties [41,42].

In summary, the synthesis method of CNFs plays a crucial role in determining their unique properties and promising potential uses. As a result, the choice of preparation conditions for the precursor material must be highly controlled.

## 2. Materials and Methods

PAN (Mw ≈ 150,000 g mol^−1^), from Sigma Aldrich, Saint Louis, MO, USA, was used as the polymer fiber precursor. As a solvent, DMF (≥99.8%), ACS Karal, was used with ρ = 0.98 g mL^−1^, and a boiling point of 153 °C.

### 2.1. PAN Fiber Preparation

**Figure 1:**
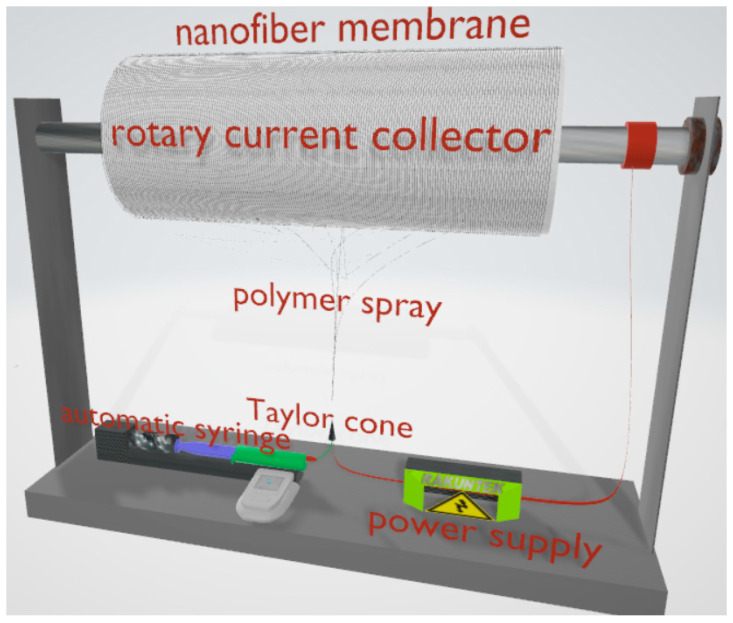
Electrospinning schema with a rotary fiber collector.

PAN was dissolved in DMF in solutions of 6, 10, 12% *w*/*w* concentrations and stirred overnight at 25 °C. Using Inovenso´s NE100 electrospinning equipment, the solution was introduced into a syringe in a pump system at a rate of 0.1 mL h^−1^. The flow variations were not studied in this article since, according to the literature, they are not significant [43]. The solution was exposed to a high voltage of 15 and 25 kV, with a distance of 15 cm between the needle tip to the solution was projected to the collecting drum. The rotational speeds were 100, 300, and 500 RPM. The PAN nanofibers produced were collected on a sheet of aluminum foil present on the collector. The electrospinning system is illustrated in Figure 1. The as prepared polymeric fiber membrane was brought to a temperature of 250 °C in an oxidative atmosphere (air) with a ramp of 5 °C min^−1^ [44].

### 2.2. PAN Fibers Characterization

Scanning Electron Microscopy (SEM) images were obtained with a Zeiss Sigma (Jena, Germany) HD VP Field Emission Scanning Electron Microscope (FE-SEM). For the measurement of the fiber diameter, images were taken at 10,000×. Using the Image-J software (version 1.53o) [45], the cross- section of 200 fibers was measured from a total of 5 groups of images from different regions of the sample. To characterize the PAN after the stabilization process, the FT-IR Nicolet™ Is™10 from Thermo Scientific™ (Waltham, MA, USA) was used to perform Fourier transform infrared spectroscopy analysis in a range of 3000–1000 cm^−1^.

Wettability was measured with contact angle using ultrapure water, employing a sessile drop mode with DataPhysics OCA 15EC. The pH of the employed water was 8.35 ± 0.49 at 25 °C, and its conductivity was 0.203 ± 0.089 μS cm^−1^. Before the $\theta_{w}$ measurement, the fiber cloth was dried for 48 h at 35 °C in a convection oven, and then for another 24 h in a desiccator. Double-sided tape was used to adhere the samples to glass slides. A Hamilton glass syringe with a maximum capacity of 500 μL and a stainless steel needle with an outside diameter of 0.53 ± 0.01 mm were used to deposit the water drops. Each drop had a volume of roughly 3.25 ± 0.18 μL. Angles $\theta_{w}$ were measured 166 ms after drop application. Using an elliptical adjustment, the right and left contact angles (given in °) of each drop were determined from the digital image, with an experimental error value of 3°. For reproducibility, ten drops were deposited on various regions on both flanks of the same fiber cloth.

To measure water uptake, the samples were placed in Petri dishes and covered with distilled water for at least 24 h to ensure complete absorption. Once the wet weight of the membranes was taken, the fiber webs were completely dried in a convection oven at 40 °C for 2 h and kept in the desiccator for at least 24 h until they were weighed again. The water uptake was obtained with Equation (1):

(1) $$Water\;uptake\;\left(\%\right)=\frac{wet\;weight-dry\;weight}{dry\;weight}\times 100$$

To measure surface porosity of the nanofiber web, an imaging protocol was carried out using the program Image-J (National Institute of Health, Bethesda, MD, USA). The protocol consists of the decomposition of the colors in RGB, selecting the channel with the best contrast out of three. The areas where the fibers appear are selected. The program excludes the darker areas where fibers are absent. In this way, the program provides the area corresponding to the empty zones (porosity). Surface porosity was compare with a gravimetric method proposed by Safari et al. [46,47]. To determine the weight of the enclosed water sample, the absorbed water at the sample surface was swiftly scraped using a paper filter and weighed to prevent evaporation. Equation (2) was used to calculate the porosity of the sample, where W_w_ is the weight of the sample containing water, W_d_ is the weight of the dry sample, and W_1_ is the weight of the sample in water.

(2) $$Porosity\;\left(\%\right)=\left(\frac{W_{w}-W_{d}}{W_{w}-W_{1}}\right)\times 100$$

## 3. Results

### 3.1. Dimensions of PAN Fibers

**Figure 2:**
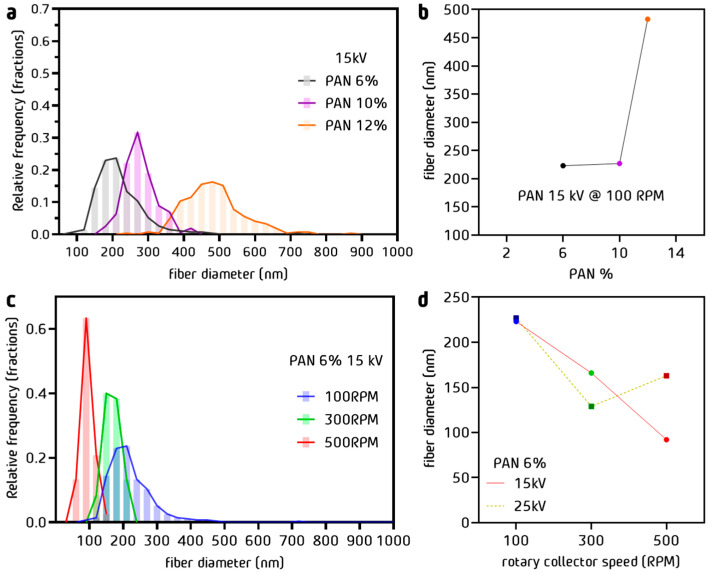
(**a**) Fiber diameter distribution and (**b**) average fiber diameter as a function of polymer concentrations at fixed drum rotation 100 RPM and fixed voltage 15 kV, black for 6% *w*/*w*, purple 10% *w*/*w* and orange for 12% *w*/*w*; (**c**) fiber diameter distribution and (**d**) average fiber diameter as a function of collector rotation at fixed 6% *w*/*w* concentration for voltages 15 kV and 25 kV, blue for 100 RPM, green for 300 RPM and red for 500 RPM.

The diameter distribution of the PAN fibers obtained by electrospinning is represented in the graphic of Figure 2a for different polymer concentrations (voltage 15 kV and drum rotation 100 RPM, fixed). Decreasing the polymer concentration from 12 to 10% *w*/*w* reduced the average diameter by almost 50% from 470 nm to 230 nm, as shown in Figure 2b. The impact of the concentration was less important between the concentrations of 10 and 6% *w*/*w*, producing a decrease from 270 to 220 nm. There was a tendency for the diminution of fiber diameter with lower percentages, and a better size distribution can also be observed in Figure 2a. The analysis of the effect of the rotation of the drum was continued with the solutions prepared at 6% *w*/*w* PAN/DMF.

The fiber´s diameter distribution is represented in the graph of Figure 2c for different drum rotation rates (fixed concentration 6% *w*/*w*, fixed voltage 15 kV). The mean fiber diameter is shown as a function of the rotation rate at two voltages (15 kV and 25 kV) at fixed 6% *w*/*w* concentration and 500 RPM. At 15 kV, we observed a linear decrease in the diameter, reaching 90 nm. At 25 kV, a decreasing trend was expected. However, due to the decompensation produced by the rotation of the collector, an increase in the diameter of the fibers occurred. In the analysis of fiber diameter, a considerable decrease in diameter was shown when the concentration of PAN/DMF varied. The decrease in diameter was caused by an increase in the collector’s rotation speed, which created a drafting force between the drum collector and the polymer jets, stretching the fiber. There is a relationship between the decrease in the diameter with the appearance of imperfections, which can be controlled by the electrospinning voltage [48].

The effect of the concentration of the polymer in solution on the average fiber size has been extensively studied [48]. This effect is explained by the increase in viscosity relative to concentration. There is an internal resistance in the structure of a polymer thread that has been compressed by an electric field. On the other hand, the increase in the electric field leads to a decrease in the diameter of the Taylor cone, a cone formed between the needle tip and where the filament starts to form (Figure 1). It is shown that at a higher rotational speed of the drum collector, a relation between the tension and concentration parameters appears, which implies an inverse effect for the diameter size.

The drum movement improved the uniformity at the expense of producing defects in the fibers. With the 25 kV voltage, it was possible to obtain a more controlled morphology by increasing the rotation speed. This means that it is possible to reduce the deformities caused by the increase in the rotation speed by increasing the electrospinning voltage, which is discussed further below.

### 3.2. Morphology of PAN Fibers

**Figure 3:**
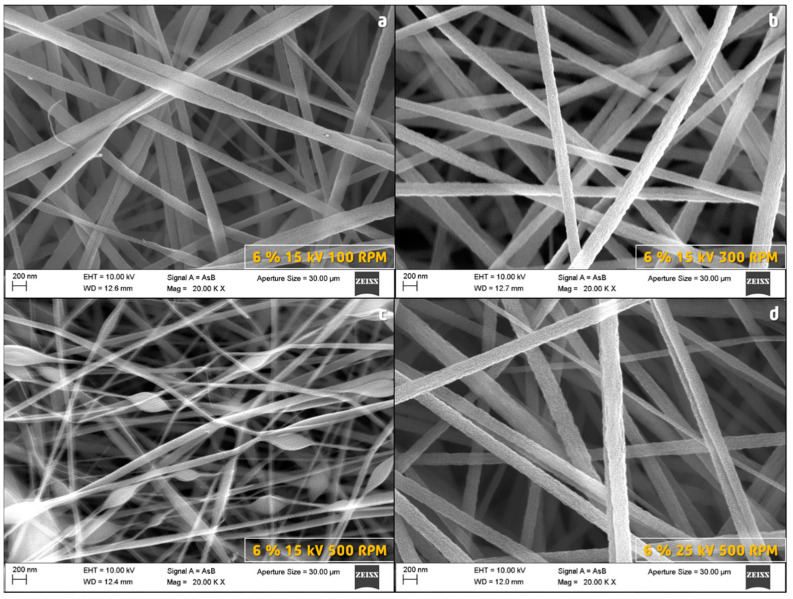
SEM images of PAN fibers prepared under different electrospinning conditions.

It was observed that the PAN fibers presented a significant change in the homogeneity of the diameter through the length of the fiber. For the same concentration of 6% *w*/*w* and voltage 15 kV, the fibers obtained at 100 RPM showed slight stretching (Figure 3a). However, the fibers prepared in similar conditions but at 300 RPM (Figure 3b) presented a better diameter homogeneity, which is due to their submission to a higher stretching force. This compensates for the possible effects of speed variation caused by fluctuations in the electric field at higher rotation rates such as 500 RPM (Figure 3c). Many diameter irregularities were observed along each fiber because the drag force produced by the collector generates non-homogeneous stretching areas. In this case, the rotation speed was probably too high and produced excessive stretching. The negative effect of the rotation at 500 RPM can be compensated by increasing the electrospinning voltage up to 25 kV (Figure 3d). This compensation was obtained because by increasing the voltage it is possible to accelerate the flow of the polymer in such a way that there is always a high feeding of fibers matching the fast rotation of the current collector (drum).

### 3.3. Stabilization Process: Chemical and Morphology Evolution

**Figure 4:**
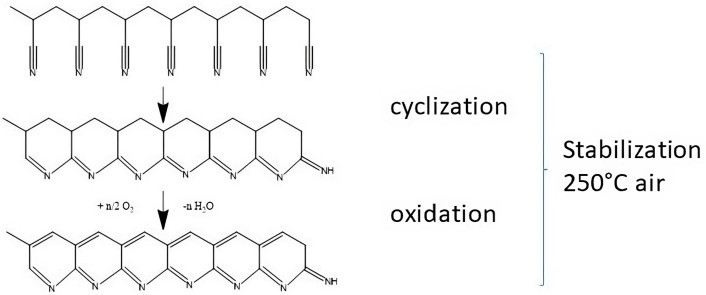
Cyclization and oxidation reaction in PAN.

**Figure 5:**
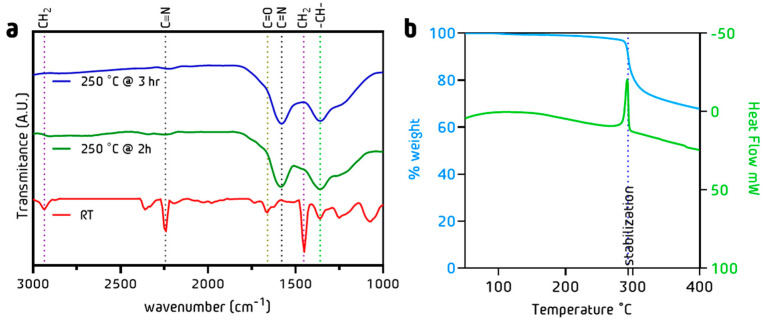
(**a**) FT-IR spectra for the stabilized PAN fibers at 200 °C 2 h and 3 h (**b**) TGA of PAN fibers under air.

To determine the dehydrogenation and cyclization degree (Figure 4), FTIR measurements (Figure 5a) were performed. The dehydrogenation process takes place in the tertiary carbon of the PAN chain. The change in the (CH_2_, CH) curve areas was quantified with Equation (3). The cyclization process takes place between the carbon of the CN and the nitrogen of the CN adjacent to the PAN structure. The change in C≡N to C=N intensities in FTIR spectra can be quantified using Equation (4) [49]. Figure 5b shows a fast weight loss at 300 °C, which corresponds to the PAN oxidation processes that result in the carbon ladder structure (Figure 4). This is followed by a steady weight reduction caused by the removal of the CH, NH, and C=N species in the polymer [50].

(3) $${\%}_{cyclisation}=\frac{I_{C=N\;}}{I_{C=N\;}-I_{C\equiv N\;}}\times 100$$

(4) $$Index_{deshydrogenation}=\frac{A_{\left(C-H\right)}}{A_{\left({\mathrm{CH}}_{2}\right)}}$$

Fibers treated at 250 °C during 2 h exhibited a dehydrogenation of 6.5%, contrary to the 13.4% shown by the 3 h treatment (Table 1). On the other hand, the cyclization degree after 2 h was 63% and 82% after 3 h [44]. This technique has been widely reported in the literature mainly using temperatures above 250 °C [50,51]. Son et al. reported a combined technique assisted by UV irradiation but with 10% less cyclization than the one reported in this work [52]. Kong et al. reported an extensive study on the impact of stabilization time using a thermal technique with similar results of 82% cyclization using higher temperatures [53].

**Figure 6:**
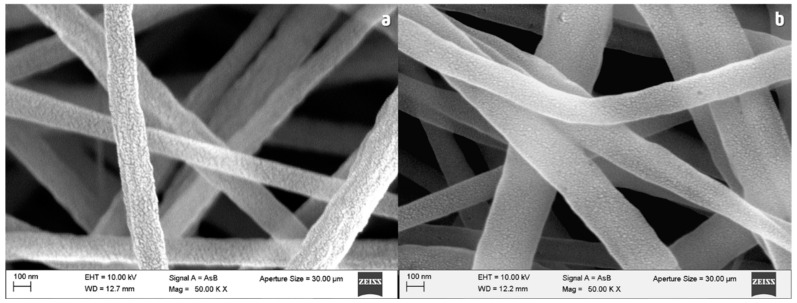
SEM microscopy of PAN fibers (**a**) before and (**b**) after stabilization for 3 h.

Figure 6a shows the morphology of PAN fibers before the stabilization process. After stabilization at 250 °C for 3 h, the surface of the fibers became smoother (Figure 6b). This effect is caused by the chemical compaction and reticulation of the polymeric network by to the cyclization reaction. On the other hand, the appearance of small pinholes can be seen, most likely due to the release of water vapor during the heat treatment.

### 3.4. Wettability of PAN Fibers

**Figure 7:**
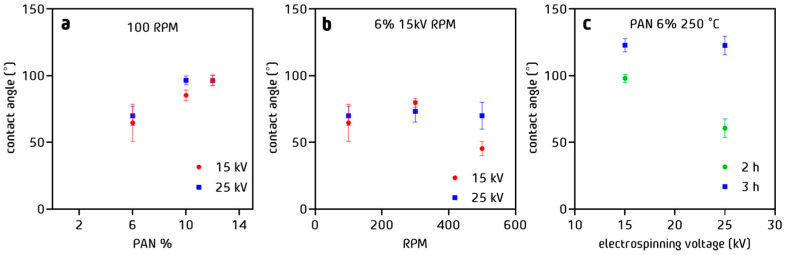
Contact angle values for the PAN fibers stabilized at 250 °C (**a**) for 2 h as a function of % *w*/*w* PAN in solution, (**b**) for 2 h as a function of drum rotation rate RPM, and (**c**) as a function of electrospinning voltage for two stabilization times.

For a constant rotation rate, an increase in the contact angle from 60° to 90° was observed when the PAN concentration increased from 6% *w*/*w* to 12% *w*/*w* (Figure 7a). The voltage (15 kV or 25 kV) had no effect. This effect has a linear impact on wettability, so it may not be entirely determined by the diameter of the fibers, which grows exponentially as a function of PAN percentage. However, a compacting effect of the fibers may be involved. On the other hand, the preparation potential did not affect the value of the contact angle. This indicates that the fibers did not present a significant change in their surface in terms of the applied potential. Regarding the effect of the rotation of the current collector (Figure 7b), there was also no appreciable effect on the value of the contact angle, suggesting that the chemical nature of the fibers is not affected. On the other hand, fibers with more hydrophobic characteristics than those reported in the literature have been obtained with 50° more than those reported by Aijaz [43]. Figure 7c shows the contact angles measured on the PAN fibers prepared at 15 kV, 6% *w*/*w*, and 100 RPM treated at 250 °C during 2 h and 3 h. Regarding the effect of the stabilization time, the fibers treated for 3 h showed higher contact angles than those treated for 2 h and became hydrophobic with contact angles above 110°. This probably corresponds to the larger cyclization degree of these fibers, confirming that the changes have an important effect on the superficial characteristics, as first observed through the SEM images.

### 3.5. Water Uptake of PAN Cloth

**Figure 8:**
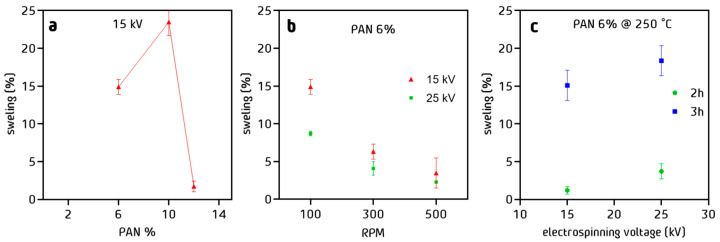
Water absorption of PAN fibers as a function of (**a**) % *w*/*w* of PAN in solution, (**b**) RPM, and (**c**) stabilization time.

Regarding to the water uptake, fibers prepared at 10% *w*/*w* PAN in solution showed a greater percentage of swelling than the fibers prepared at 12% *w*/*w* (Figure 8a). This suggests that the structure and compaction of the fibers have an important effect on the amount of water that the fiber can retain. It can be concluded that the water uptake is inverse to the diameter. The larger the diameter, the less space the material has to swell. The decay in water uptake as a function of the rotation speed was also observed (Figure 8b). The fibers compaction plays an important role in the amount of liquid retained during the water uptake experiments. Fibers with a more compact arrangement are less favored for this purpose than fibers with more disordered arrangement. After a stabilization process at 250 °C for 2 h (Figure 8c), a significant decrease of the water uptake was observed, whereas after stabilization for 3 h, the swelling values corresponded to those observed before the thermal treatment.

### 3.6. Porosity of PAN Cloth

**Figure 9:**
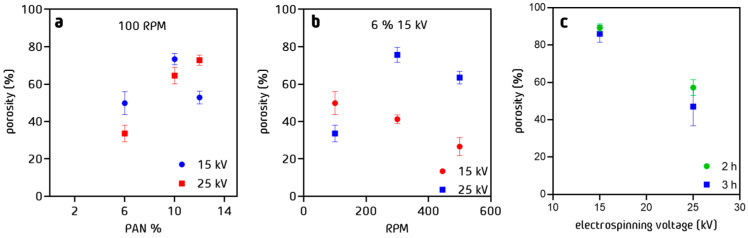
Porosity % of PAN cloth fibers measured by imaging method as a function of (**a**) % *w*/*w* of PAN in solution, (**b**) RPM, and (**c**) stabilization time process at 250 °C.

Porosity studies show that increasing the percentage of PAN in the electrospinning solution and therefore fiber diameter, increases the level of surface porosity of the PAN fiber web before stabilization (Figure 9a). However, this effect does not allow the compaction of the fibers, probably due to a repulsion effect of the fibers during the projection of the threads during electrospinning. It was observed that with a potential of 25 kV, more compact and less porous structures were obtained. This is due to the increase in the tension on the fiber at the time of the electrospinning process, compensating for the repulsion of the fibers by joining them together. Regarding the effect of rotation (Figure 9b), it has been observed that the fibers prepared with 15 kV tension and a percentage of 6% *w*/*w* show a decrease in surface porosity with respect to the increase in rotation speed, suggesting that the stretching of the fibers caused by the rotation of the drum helps the fiber compaction. As previously demonstrated, the increase in electrospinning voltage compensates for the rotation effect, increasing fiber surface porosity up to a maximum of 80% at a rotation of 300 RPM. However, above this value of rotation, a decrease in surface porosity was observed, once again resuming the compaction of the fibers. Considering the impact of the stabilization process (Figure 9c), there is no significant change between the samples treated for 2 or 3 h, since the fibers only change their internal structure but not the external one.

**Figure 10:**
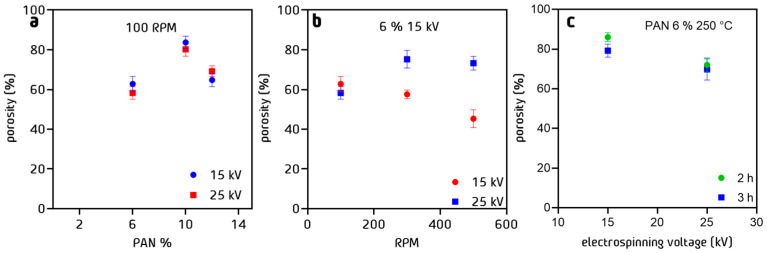
Porosity % of PAN cloth fibers measured by the gravimetric method as a function of (**a**) % *w*/*w* of PAN in solution, (**b**) RPM, and (**c**) stabilization time process at 250 °C.

To compare the results obtained by image analysis, the gravimetric method was applied (Figure 10), providing a quantitative measure of porosity. It directly calculates the percentage of porosity based on the weight change before and after saturating the material with a fluid. We used both methods in conjunction to gain a more complete understanding of the PAN nanofiber’s porosity characteristics. The differences between each method were maintained within a value of 3% to 5% maximum, which suggests that the results obtained with the previously proposed method can be used in polymeric fibers with similar characteristics. The difference is not significant, because the PAN nanofibers do not have fine pores or irregular pore shapes, which means that the porosity being measured is the porosity between nanofibers, not within each of the nanofibers.

## 4. Discussion

PAN fibers with around 90 nm diameter were successfully obtained by adjusting the electrospinning parameters. The concentration of PAN in the electrospun solution plays an important role since fibers below 100 nm can be obtained with concentrations below 10% *w*/*w*. On the other hand, increasing the speed of rotation allows for diameter reduction, given the stretching of the polymeric fibers during electrospinning as demonstrated in the SEM analysis. The voltage applied between the nozzle and the drum collector also plays a role with a decrease in diameter observed increasing the voltage. However, it is important to match the rotation rate of the drum collector to the voltage of electrospinning.

The drum collector rotation rate mechanically pulls the fibers and the voltage of the electrospinning controls the flow of the polymer solution from the injection tip to the drum. If the matching is not good, heterogeneous diameters or agglomerated particles appear.

The chemical structure and the degree of cyclization during the stabilization process at 250 °C strongly depend on the treatment time. Between 2 h and 3 h, an improvement of 20% in the cyclization process was observed, as well as in the dehydrogenation process. Regarding the morphological changes observed in the fibers, changes can be seen in the surface of the fibers, from rough with some textural irregularities to a more compact and smooth structure.

To allow the greatest contact of effluent with the fiber, the diameter of the fiber should be the smallest. These effects on the surface are given by the formation of ladder links that are formed during the reaction, helping to compact the polymeric structures. Regarding wettability, an increase in the contact angle values is observed as a function of the percentage of PAN. The higher the fiber diameter, the higher the hydrophobicity of the material. It was observed that the rotation of the collector did not have a considerable effect on the wettability of the fibers. The swelling properties of the fibers were considerably affected by the percentage of PAN, showing a maximum level of swelling of 10%. The water uptake decreased to very low values when PAN was electrospun at 12% *w*/*w*. The fiber diameter presented an inverse behavior, which suggests that the space occupied by the larger diameter fibers hampers the absorbent properties of the material. In the same way, the compaction of the fibers affects the swelling of the fibers. By increasing the compaction of the fibers, the free spaces remain occupied, thus reducing their absorbing capacity. The porosity of PAN cloth is proportional to the diameter of the fibers. The greater the diameter, the greater the tendency to separate from each other, which increases the porosity.

## 5. Conclusions

This study focuses on synthesizing and characterizing PAN fibers as high-quality precursors for carbon fibers, potentially used in fuel cell cathodes or metal–air batteries. PAN concentration in the electrospinning solution impacts fiber diameter, with concentrations below 10% *w*/*w* yielding diameters less than 100 nm. Increased rotational speed during electrospinning allows better control of fiber shape. The study also examines the effect of a 250 °C stabilization technique on PAN fiber chemical structure, revealing a 20% enhancement with a 3 h treatment. Contact angle values increase with higher PAN percentages, and swelling properties peak at 10% *w*/*w* PAN, decreasing at higher concentrations. Greater fiber diameter leads to increased porosity, as fibers separate. Additionally, 90 nm diameter PAN fibers were obtained under 6% *w*/*w*, 25 kV, and 500 RPM. Overall, the study highlights the importance of electrospinning settings to produce PAN fibers with suitable properties for use as carbon fiber precursors contributing to the knowledge of nanofiber fabrication for sustainable energy systems.

## Figures and Tables

**Table 1:** Dehydrogenation and cyclization index as function of time for PAN fibers.

Process		2 h	3 h
Dehydrogenation	${\mathrm{CH}}_{2}$	6.48	13.42
	CH		
Cyclization	C≡N	62.96	81.94
	C=N		

## Data Availability

The data that support the findings of this study are available from the corresponding author upon reasonable request.
